# On-chip fabrication of tailored 3D hydrogel scaffolds to model cancer cell invasion and interaction with endothelial cells

**DOI:** 10.1063/5.0227135

**Published:** 2024-12-03

**Authors:** Federico Cantoni, Laurent Barbe, Ananya Roy, Grzegorz Wicher, Stina Simonsson, Karin Forsberg-Nilsson, Maria Tenje

**Affiliations:** 1 Department of Materials Science and Engineering, Uppsala University, Uppsala, Sweden; 2 Science for Life Laboratory, Uppsala University, Uppsala, Sweden; 3 Department of Immunology, Genetics and Pathology, Uppsala University, Uppsala, Sweden; 4 Department of Medicinal Chemistry & Cell Biology, Institution of Biomedicine, Department of Clinical Chemistry, Gothenburg, Sweden; 5 Region Västra Götaland, Sahlgrenska University Hospital, Department of Clinical Chemistry, Gothenburg, Sweden

## Abstract

The high mortality associated with certain cancers can be attributed to the invasive nature of the tumor cells. Yet, the complexity of studying invasion hinders our understanding of how the tumor spreads. This work presents a microengineered three-dimensional (3D) *in vitro* model for studying cancer cell invasion and interaction with endothelial cells. The model was generated by printing a biomimetic hydrogel scaffold directly on a chip using 2-photon polymerization that simulates the brain's extracellular matrix. The scaffold's geometry was specifically designed to facilitate the growth of a continuous layer of endothelial cells on one side, while also allowing for the introduction of tumor cells on the other side. This arrangement confines the cells spatially and enables *in situ* microscopy of the cancer cells as they invade the hydrogel scaffold and interact with the endothelial layer. We examined the impact of 3D printing parameters on the hydrogel's physical properties and used patient derived glioblastoma cells to study their effect on cell invasion. Notably, the tumor cells tended to infiltrate faster when an endothelial cell barrier was present. The potential for adjusting the hydrogel scaffold's properties, coupled with the capability for real-time observation of tumor-endothelial cell interactions, offers a platform for studying tumor invasion and tumor–endothelial cell interactions.

## I. INTRODUCTION

Cancer is one of the leading causes of death worldwide, and despite extensive molecular studies, some cancers lack treatment.^1,2^ A key aspect in developing effective therapeutics for tumors is the understanding of invasion and recurrence mechanisms, since these account for around 90% of cancer deaths.^3,4^ Especially in glioblastoma (GBM), rapid local invasive growth of cancer cells after surgical resection is one of the reasons the tumor remains incurable. Despite tumor cell invasion and intravasation being essential steps in tumor spreading, many of the driving biological mechanisms involved are still poorly understood.^5,6^

A reason for the limited success rate of developing oncologic treatment is that animal models in preclinical trials can have poor prediction power of human physiology. Although animal models have represented an essential tool to progress the understanding of human biology and disease mechanisms, these same models present several drawbacks including time-consuming development, high complexity, limited access to the biological event, and poor predictability of the human response to new treatments.^7,8^

*In vitro* models, such as two-dimensional (2D) cell mono or co-culture on flat and rigid surfaces, provide a user-friendly and cost-efficient platform for high throughput screening of new drug candidates and fundamental biology studies.^9^ The implementation of a porous membrane in 2D systems creates an upper and lower compartment introducing multicellular cultures for cell migration and cell–cell interaction studies.^10^ However, such a strategy still poorly replicates reproducible gradients,^11^ has limited membrane transparency, and fails to recapitulate the 3D native environment and hampers the cell–cell interactions leading to aberrant cell behavior.^12,13^

In an attempt to advance *in vitro* models and bridge the gap with the high complexity of animal models, microphysiological systems (MPS) have been developed to recreate biologically relevant environments in miniaturized devices.^14,15^ In MPS, microfluidics provides dynamic control over the cellular microenvironment while spatially and temporally confining fast and random cell events, such as cell invasion, to enable the investigation of biological mechanisms.^16–18^ On such platforms, 3D matrices engineered to resemble the extracellular matrix (ECM) environment, either based on hydrogels^12^ or porous structures,^19,20^ can be coupled with 3D cell cultures.^8,21,22^ The introduction of 3D culture served to understand fundamental interactions between the cells and the surrounding microenvironment.^12^ This includes the cell-ECM as well as the cell–cell interactions. Natural-based hydrogels, such as fibrin and collagen, have been largely used for the self-assembly of human vascular endothelial cells (HUVECs) on-chip to recreate 3D vascularized constructs in which encapsulated cancer cells or spheroids are integrated to study invasion and intravasation mechanisms.^23–25^ Despite ensuring a vascularized network, the poor control over the self-assembling of cells hampers the system's reproducibility, thus limiting comparative studies. In contrast, compared to natural-based hydrogels, synthetic and hybrid materials provide greater control over material properties,^26^ overcome batch-to-batch variation, and have the potential to recreate the architecture of tissues. Techniques including gas foaming,^27^ electrospinning,^28^ and particulate leaching^29^ have been investigated to tune matrix porosity.^26–28^ Such strategies successfully increase the overall hydrogel porosity, but little or no spatial control over the mechanical properties and porosity distribution negatively affects cell development and invasion.^30^

Among the different 3D printing technologies today, 2-photon polymerization (2PP) has emerged as a reliable strategy to obtain scaffolds with subcellular feature sizes and resolution suitable for mimicking biologically relevant human tissue-like environments.^31–41^ The high-spatial control and possibility to build complex architectures with 2PP makes it possible to fabricate structures that accurately mimic biologically relevant environments for studying tumor invasion and interactions with endothelial cells.^20,34,42,43^ The poor processability of hydrogel-based inks, due to swelling in cell media, and poor mechanical properties has, however, hampered the use for structures with sub-100 *μ*m resolution features, favoring resin-based materials instead. However, the resin-based scaffolds scatter visible light reducing imaging quality, present around three orders higher Young's modulus compared to human soft tissues,^44^ and display high autofluorescence in a wide visible spectrum.^45^ Moreover, the lack of degradable moieties in these resin-based scaffolds not only hinders the invasion and migration of tumor cells within the scaffold, which is crucial for mimicking a tissue-like environment, but also limits the potential for dynamic remodeling of the scaffold over time. This impairment reduces suitability for long-term biological studies and applications of resin-based scaffolds where tissue integration and natural degradation are essential.

Here, 2PP is used to 3D print on-chip a hydrogel scaffold hosting tumor cells and endothelial cells. The direct integration of the hydrogel scaffold on-chip facilitated the handling of the coculture while reducing the 2PP printing time to 20 min. The conceived scaffold design mitigated the hydrogel swelling and ensured the tuning of the hydrogel properties with high spatial accuracy (40 *μ*m) to study single-cell invasion in a soft-tissue-like environment (Young's modulus around 1 kPa) in the presence of endothelial cells. The physical confinement of the tumor cell invasion combined with the vicinity to the endothelial monolayer allowed tracking of multiple and simultaneous cells by confocal microscopy, from the initial seeding until the end of the experiment. The proposed model was validated with glioblastoma cells for their high invasiveness and interaction with endothelial cells. More specifically, we have focused on the invasion and interaction of patient-derived GBM cells with an endothelial barrier over 5 days. This new platform provides a versatile tool where the scaffold properties can easily be tuned to investigate cell invasiveness of different tumor types in their respective soft tissue-like environment while providing continuous monitoring of tumor cell invasion and interactions with the endothelial cells.

## II. RESULTS AND DISCUSSION

Since the ECM plays a key role in determining cell responses, the capability to tune the physical properties of a hydrogel is expected to improve *in vitro* models for cell invasion. However, the tuning of hydrogel properties at the cellular length scale inside a microfluidic device remains a technical challenge. In particular, in an attempt to replicate soft tissues, hydrogels with Young's modulus around 1 kPa generate constructs with poor mechanical stability that are prone to high swelling due to the low cross-linking density.^32,39^ Additionally, a major challenge with current co-culture MPS for studying cell–cell interaction is the difficulty of positioning different cell types within a distance from each other to be compatible with time-lapse microscopy. Cell tracking, for instance, using confocal imaging over large culture volumes for several days, can generate an extensive amount of data, but lacks the speed necessary to monitor rapid cellular events.^41^

**FIG. 1.**
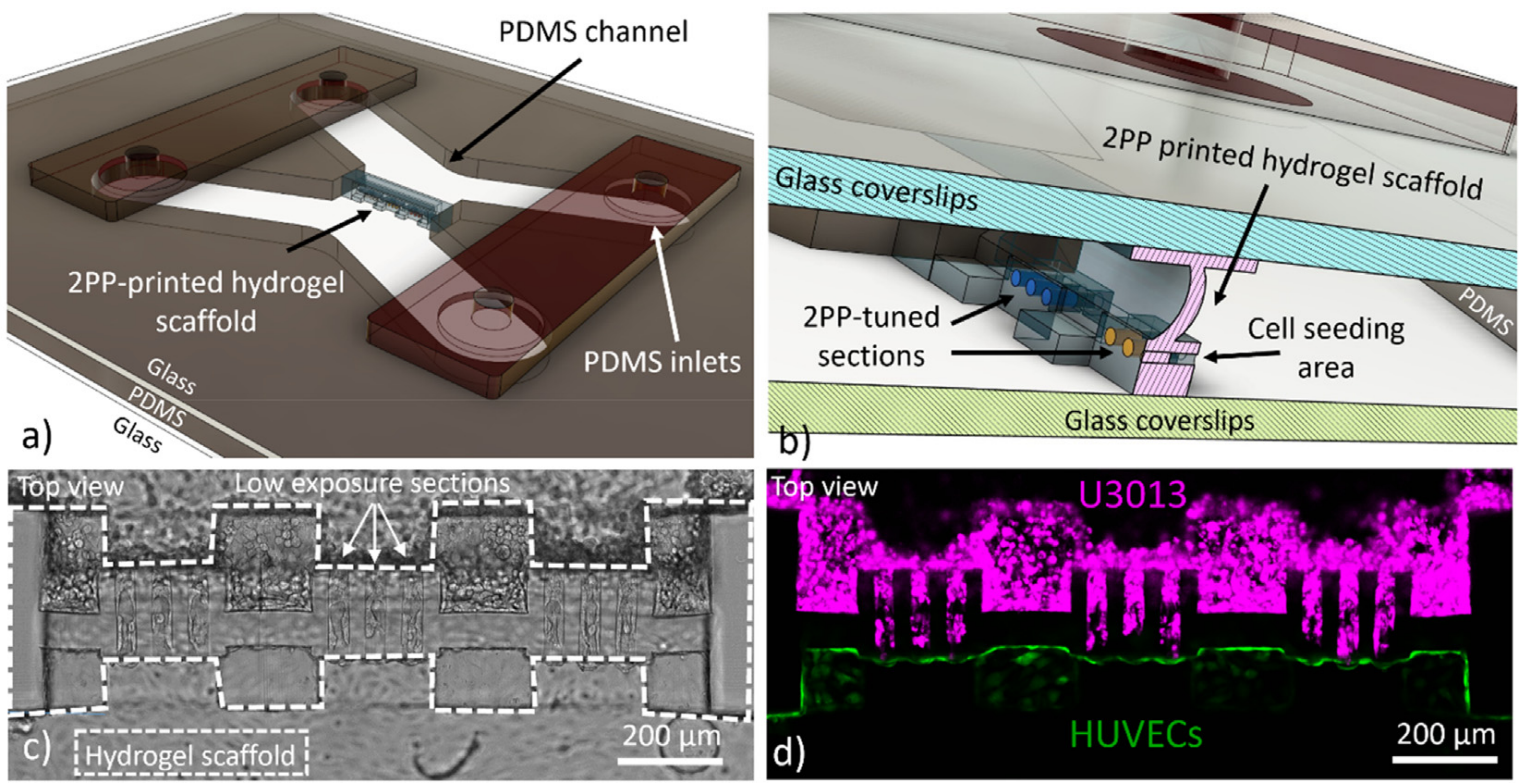
The computer-aided design (CAD) of the microfluidic chip with the hydrogel scaffold separating the vascular side (HUVEC) from the channel with the tumor cells (U3013), (a). Magnification of the CAD design to display the details of the hydrogel scaffold and the microfluidic chip components (b). Brightfield, (c), and fluorescent, (d), image of the magenta-labelled U3013 cells (top channel) and green-labelled HUVECs (bottom channel) cultured on the opposite walls of the hydrogel scaffold (Leica SP8, 10× and NA 0,3 objective).

In this study, we propose a strategy to benefit from the high spatial resolution of 2PP 3D printing to generate on-chip a hydrogel scaffold with tunable physical properties, as shown in Figs. 1(a) and 1(b) and supplementary material, Fig. 1. By modulating the laser power during printing, we embedded low energy-crosslinked hydrogel sections within a mechanically stable scaffold, both defined in a gelatin-based ink, as shown in Figs. 1(c) and 1(d). Such scaffold configuration enabled the screening of different 3D printing parameters for tumor cell invasion and interaction with HUVECs, as shown in Figs. 1(c) and 1(d). The scaffold was directly 3D printed on-chip and designed to separate the two communicating microfluidic channels, including either tumor or endothelial cells, shown in Figs. 1(a)–1(d). The separation of the device into two seeding chambers allowed the introduction of tumor cells after the formation of a uniform endothelial monolayer. The fabrication directly inside the chip ensured good alignment between the hydrogel scaffold and the larger structures of the microfluidic channels while preventing sources of contamination or potential handling damages. The scaffold design allowed the integrity and function of the hydrogel structure by confining the hydrogel deformation due to swelling to dedicated sections, supplementary material, Video 1. The cell invasion confined in a small volume simplified the monitoring of events compared to *in vivo*, *ex vivo,* and spheroid cell cultures where tissue penetration can be limited and large z-stacks are typically required.^9,46^ In our work, glioblastoma cells were used to validate the proposed model due to their highly invasive nature and their well-documented ability to interact with the surrounding vasculature.^47,48^

### A. 3D printing parameters influence the scaffold's physical properties

2PP 3D printing enables the accurate control of geometry and energy dose, which can be used to easily tune the architecture and physical properties of a hydrogel structure.^31,32^ To benefit from these capabilities, we established a laser power interval (40–90 mW) for fabricating the hydrogel and perform cell invasion studies. Since energy dose is correlated with the cross-linking density, the equilibrium swelling, diffusivity, and mechanical properties were measured and analyzed.^49^

**FIG. 2.**
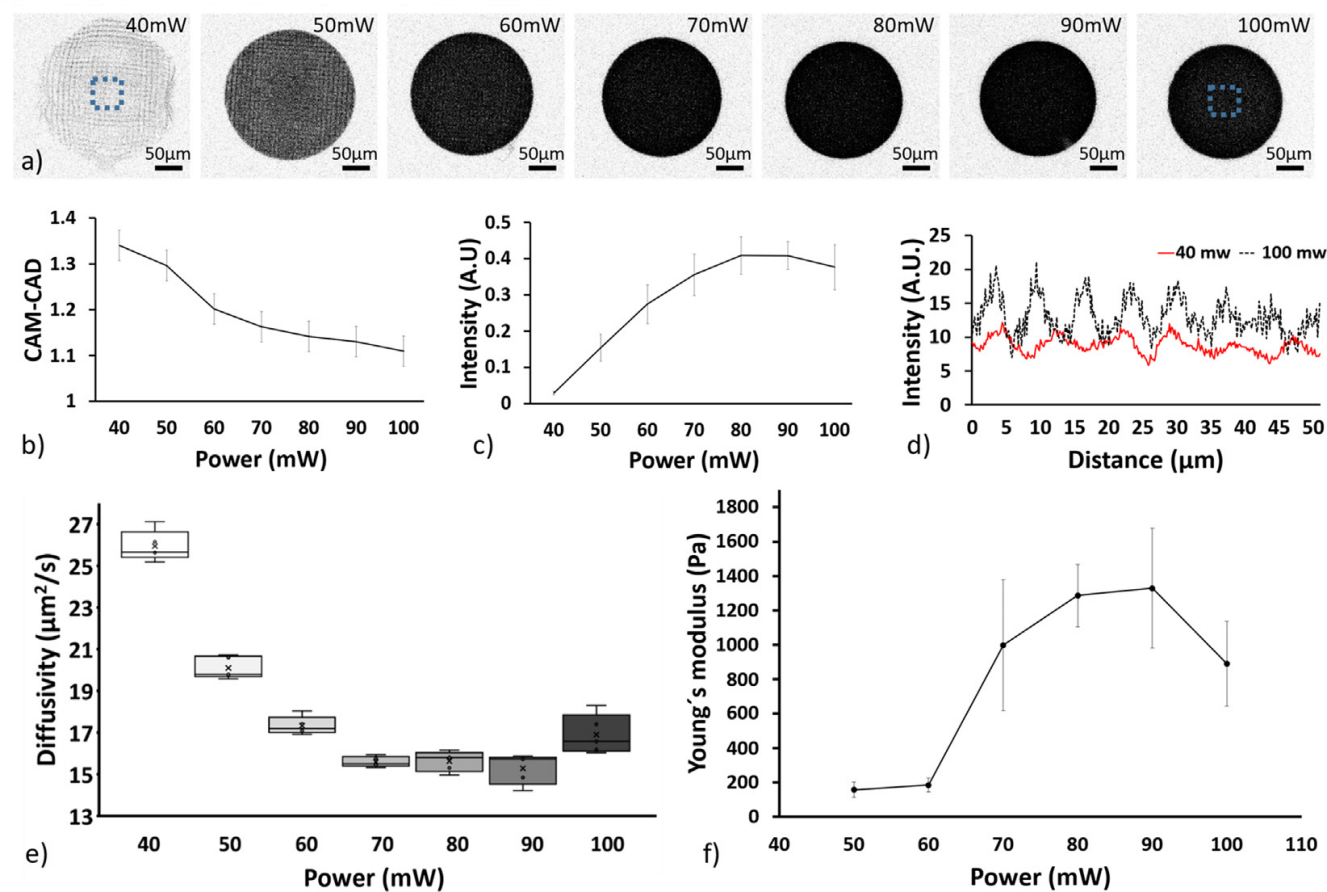
Fluorescence confocal images of the laser power screening samples (disk) retained on the glass substrate, top view (Leica SP8, 10× and 0.3 NA objective). Look up table of the images were inverted to improve visualization (a). Average intensity (b) and CAM-CAD mimicry (c), of the top surface of the hydrogel disk obtained for the screened energies (n = 3, error: standard deviation). Intensity profile of the fiber constituting the hydrogel structure for 40 and 100 mW (d). The analyzed area is highlighted in the dashed rectangle shown at 40 and 100 mW in (a). Diffusivity of 70 kDa FITC-dextran in the 2PP hydrogel after 24 h (n = 5, error: standard deviation) (e). The Young's modulus of samples obtained with the screened energies, (n = 3 with 4 measurements per sample, error: standard deviation) (f). Data could not be obtained for the 40 mW samples due to the detection limit of the instrument.

The laser power was screened between 30 and 100 mW with constant 0.5 *μ*m hatching and 3 *μ*m slicing layer intervals, as shown in Fig. 2(a). The hatching and slicing layer intervals were not varied as our previous work has confirmed these to be optimal for hydrogel 2PP.^31^ To ensure uniform properties of the structures, the woodpile setting was chosen. In addition, the “top-down” printing mode was preferred to the “bottom-up mode” to ensure a higher printing fidelity.^32^ All the structures were incubated for 24 h in cell culture media to allow equilibrium swelling after removal of the non-crosslinked hydrogel precursor solution. Despite being visible after printing, the sample obtained with 30 mW was not retained on the glass surface after the removal of the non-crosslinked hydrogel precursor solution, as shown in supplementary material, Fig. 2. All work with the samples, including removal of non-crosslinked hydrogel and imaging, was conducted on-chip to reduce direct manipulation of the hydrogel scaffold with a reduction of possible damages and contaminations.

Even if the 2PP 3D printing process ensures high cross-linking confinement in the laser voxel, the hydrophilic nature of gelatin causes swelling of the structure when placed in aqueous solutions. The structure swelling reduces printing fidelity deforming the structure and affecting the scaffold function.^50^ High degree of deformation might cause inter-fiber detachment compromising the continuity of the scaffold, as shown in Fig. 2(a). Both the fiber-like 3D printing process and the hydrogel swelling need to be considered when designing and working with 2PP defined hydrogel structures.

The swelling behavior for the screened powers was investigated, shown in Fig. 2(b). The top of the disk was analyzed to reduce the influence of the glass–substrate constrain. The computer-aided manufacturing and the computer-aided design ratio (CAM/CAD) was taken as a qualitative measure of how accurately the design could be replicated and it was seen to decrease as the power increased, as also observed in previous studies.^49^ Below 70 mW, small changes in power resulted in large changes in the CAM/CAD. In contrast, when powers above 70 mW were used, we only observed a small influence of the power on the sample swelling. Interestingly, a similar trend, but with the opposite slope, was observed for the hydrogel fluorescence intensity, as in Fig. 2(c). Here, a higher cross-linking density correlates with a higher amount of fluorescence photo-initiator being trapped in the hydrogel scaffold.^51^ The 40 mW sample showed both the highest disk surface area and the lowest structure circularity, associated with poor cross-linking. The weakly connected fibers lead to high swelling and an increase in sample volume. The 40 mW hydrogel structure also displayed the highest intra- and inter-fiber porosity, due to the lowest cross-linking density and fiber adhesion, respectively, as in Fig. 2(d). No deviation from circular shape was observed for energies above 40 mW, as shown in Fig. 2(a). A comparison of the fiber size between the sample obtained at 40 and 100 mW laser powers showed an increase in diameter for the lowest energy as a consequence of the higher swelling, as in Fig. 2(d). In addition, the intensity profile of the fiber for both screened powers displayed a gradient from the core to the edge of the fiber, associated with the energy distribution of the voxel.^31^

A similar trend as the fluorescence intensity was observed for the stiffness of the 2PP hydrogel, as measured by nanoindentation on circular 1 mm diameter disks. For energies between 50 and 80 mW, a higher Young's modulus was observed as the laser power increased, but after reaching a plateau at ∼80–90 mW, a decrease in the mechanical properties was observed, shown in Fig. 2(f). The 40 mW samples were not measured due to the detection limit of the instrument and the high mobility of the hydrogel fibers where the low inter-fiber cohesion prevented stable contact between the nanoindenter probe and the scaffold surface. The measured Young's modulus was in the same order of human brain tissue.^52,53^

The effect of the laser power on molecule diffusivity was investigated by fluorescence recovery after photobleaching (FRAP) of 70 kDa FITC-dextran molecules, as in Fig. 2(e). As expected, the diffusivity displayed the inverse trend of the mechanical properties of the sample with a decrease as the laser power increased up to 70 mW, where a plateau was reached. The observed relation between laser power and diffusivity is associated with the decreased porosity of the hydrogel as the energy dose increased.

Interestingly, the sample obtained with 100 mW laser power displayed a lower fluorescence intensity and mechanical properties while displaying an increase in diffusivity. The lower fluorescence intensity might result from fluorophore bleaching due to the high laser power during fabrication. However, the drop in diffusivity and mechanical stability was associated with excessive energy resulting in polymer chain degradation^49^ or inactivation of the crosslinker^54^ leading to the formation of a less dense and in turn, weaker network.

From the measurements of the physical properties of the hydrogel, it was concluded that at 70 mW most of the available crosslinker and polymer side groups were reacted, inducing only small differences in material properties when higher energies were used. In contrast, below 70 mW, the lower the power, the less the amount of cross-linking points, leading to a lower Young's modulus with a higher swelling and diffusion.

These results show that the properties of hydrogel structures can be fine-tuned by varying the laser power while keeping the remaining 3D printing parameters constant. Especially, in proximity of the cross-linking threshold, the physical properties of the 3D structures change drastically with small variations in the energy dose. Consequently, we established that laser power from 70 to 90 mW is suitable for the fabrication of the hydrogel scaffold section with structural function, while laser powers between 40 and 70 mW were chosen for the parts of the scaffold dedicated to the cell invasion screening.

### B. On-chip fabrication of tailored hydrogel scaffolds

When guiding the invasion of cells in a hydrogel structure, it is important to study what role the hydrogel properties play. In this study, different physical properties of the hydrogel were obtained by tuning the fabrication parameters, as discussed in Sec. II A. In particular, the laser dosage used correlated with cross-linking density and consequently to swelling and mechanical stiffness, as shown in previous studies.^49^

Since the printed scaffold is constrained inside the microfluidic channel, the hydrogel swelling will result in scaffold deformation, compromising the surface for cell seeding and compressing the low-energy sections. In addition, the poor mechanical properties of the areas defined with lowest energies (30–50 mW) hamper the fabrication of standalone structures capable of tolerating the non-crosslinked precursor solution removal and cell media exchanges. It is therefore critical to avoid swelling while ensuring an appropriate mechanical property of the 3D scaffold. This can be achieved by either ensuring a high cross-linking degree or using synthetic polymers. However, these approaches present the drawback of limited permeability and low biomimicry lacking cell recognition and adhesion sites, respectively.^33^

**FIG. 3.**
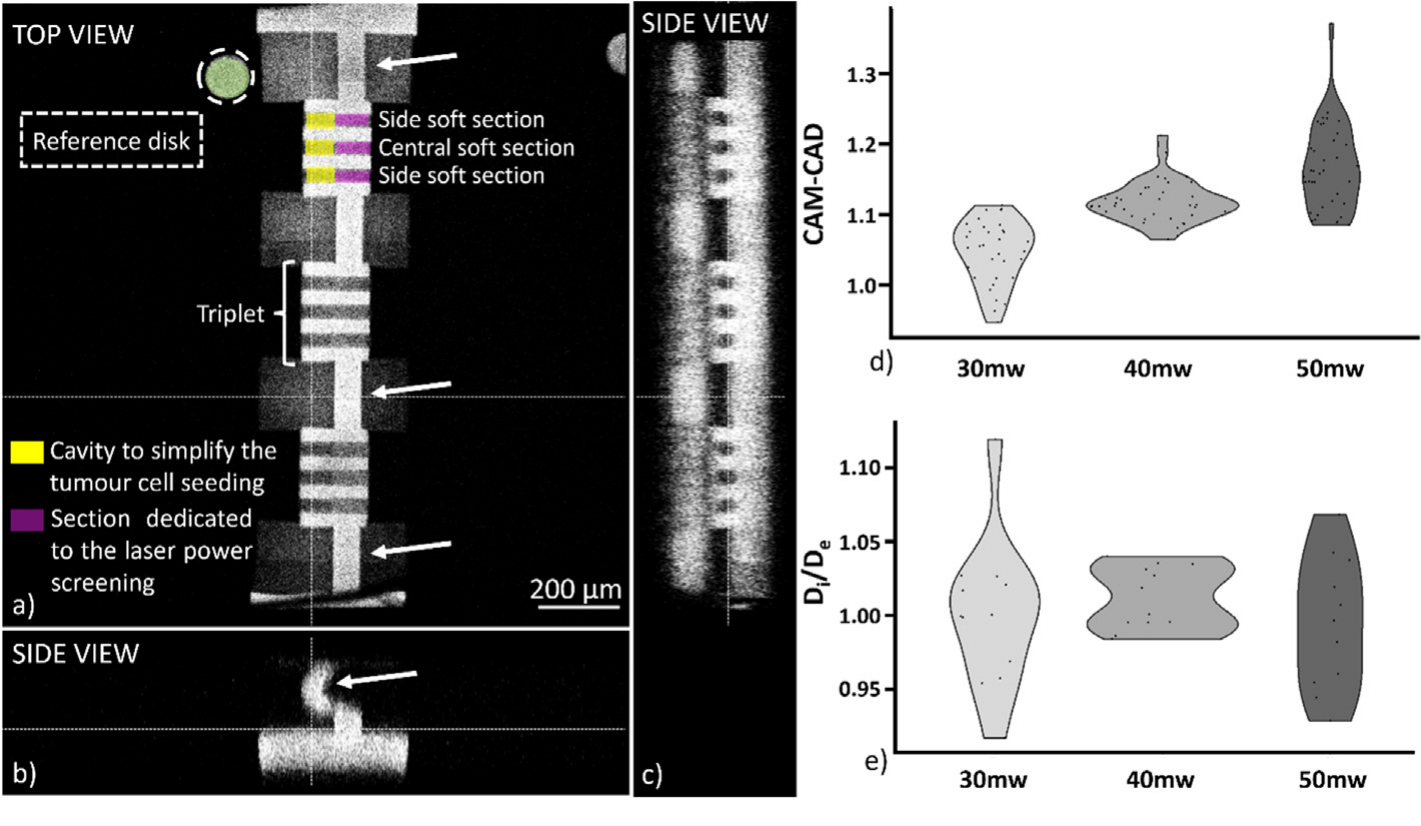
Orthogonal views from a confocal microscope stack (Leica Sp8, objective: 10×, NA 0.3) of the 3D printed hydrogel scaffold. Each microfluidic device presented sections grouped in triplets. The reference disk (green) allowed to break the structure symmetry and correlate the printing power of each triplet. White arrows indicate constructs where the deformation due to swelling is concentrated. (a)–(c). CAM-CAD mimicry along the invasion section for the three different screened energies 30, 40, and 50 mW (n = 30), (d). Ratio between the inner diameter of the soft section in the center and the soft sections on the side of each triplet (n = 10), (e).

Here, we propose to combine regions of high cross-linking, as mechanical support, together with regions of lower cross-linking that provide invasion paths for the cells. The scaffold design accounts for the hydrogel swelling by implementing thin and curved walls in which the structure deformation can be concentrated, thereby preserving the areas dedicated for the invasion assay. To evaluate optimal design of the soft regions, sections of 40 *μ*m diameter and 75 *μ*m length at the base of the scaffold were exposed with laser powers ranging from 30 to 50 mW. These sections covered the entire width of the structure and had a cavity defined on one end, to simplify tumor cell seeding, shown in Figs. 3(a)–3(c). The full structure was 3D printed within 20 min enabling up to five devices to be fabricated during the 3 h working window of the hydrogel precursor solution.

After printing and equilibrating the structures in cell media for 24 h, the CAM/CAD mimicry of all the sections obtained with the tested powers was measured via confocal microscopy to investigate what effects the hydrogel swelling had on the geometry of the structure. The fluorescence intensity difference between the areas printed with different powers allowed us to distinguish the sections of the structure, shown in Fig. 3(a). All the tested powers displayed CAM/CAD values close to 1, with a slight increase in the inner diameter of the invasion channels as the power increased, shown in Fig. 3(d). This shows that the regions of higher cross-linking fulfilled their purpose to provide mechanical support and that the design implemented reduced structure deformations.

Possible non-uniformities between the soft section in the center and the outermost soft sections of each triplet were analyzed by observing differences between the section diameters, as in Figs. 3(a), 3(c), and 3(e). No significant difference was observed between the central channel and the side channels of the triplets. Similarly, eccentricity values displayed no notable change between the screened powers. However, the sections did not present a perfect circularity, with an elongation along the z-axis, as in supplementary material, Fig. 3, associated with the more effective swelling compensation in the z-direction of the design compared to the x–y direction, shown in Fig. 3(c). All the soft sections displayed a similar geometry after the scaffold swelling, ensuring optimal seeding for the cell invasion study. The developed scaffold design enabled on-chip integration of a mechanically stable hydrogel structure with sections having different physical properties by tuning the laser power.

### C. The hydrogel scaffold supports cell culture

**FIG. 4.**
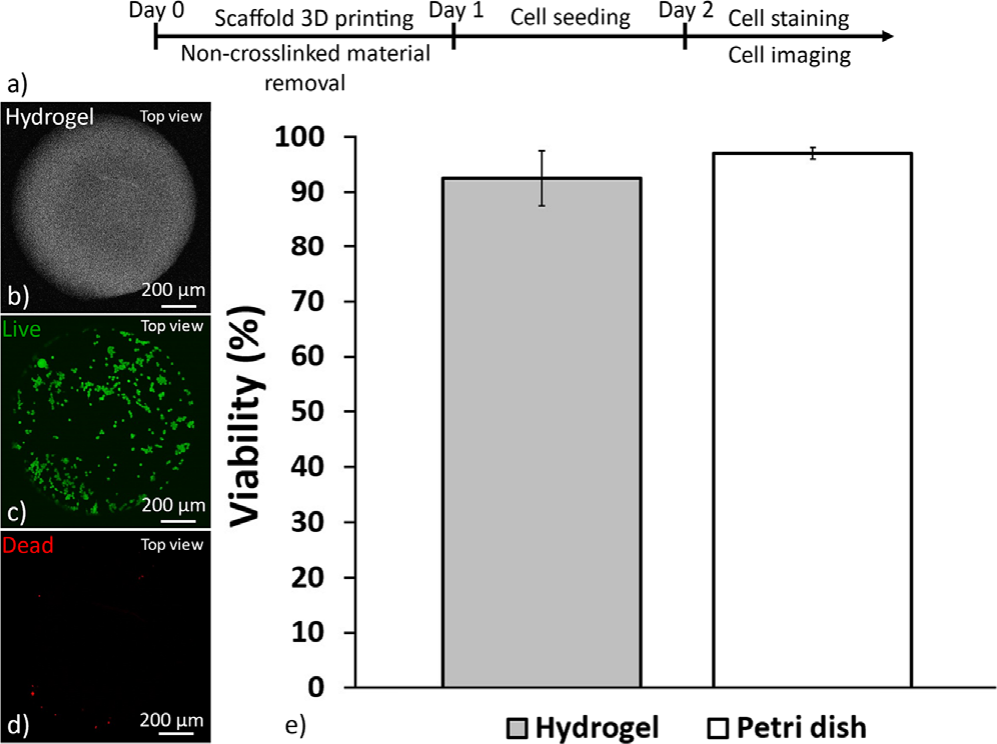
Biocompatibility of 2PP hydrogel for a 2D culture of U3013 cells. Scheme showing the hydrogel disk 3D printing and the U3013 culture protocol (a). The cells were cultured on a hydrogel disk 3D printed inside a microfluidic chip (b), stained for live (c), dead (d), and staining after 24 h of culture (Leica SP8, 10×, NA 0.3 objective) (d). Viability of U3013 cells cultures on the 2PP hydrogel and on the well plate after 24 h of culture (n = 3, error: standard deviation) (e).

Cell survival rate is one of the fundamental factors to consider when culturing cells on a new substrate. A recent study from our group showed that human umbilical vein endothelial cells (HUVECs) attach, proliferate, and spread on 2PP 3D printed hydrogel structures obtained with the same ink used here.^31^ In the current study, we wanted to evaluate the cytocompatibility of the hydrogel with tumor cells. Patient-derived GBM cells (U3013) were cultured on top of a printed hydrogel disk, according to a previously published protocol,^31^ as shown in Figs. 4(a)–4(c). The hydrogel structure was defined using 90 mW laser power to ensure a stable surface with no swelling for smooth cell seeding. U3013 cells were cultured on a laminin-coated tissue culture plate as a control. No significant differences were observed between the cell culture on the hydrogel and on the well plate surface, indicating that the hydrogel did not have a negative impact on cell viability after 24 h. Both conditions displayed cell viability above 90% after 24 h, as shown in Fig. 4(d). Consequently, the hydrogel resulted in a suitable substrate for the co-culture of HUVECs and U3013 cells.

### D. Tuned hydrogel properties affect cell invasion

The main goal of this work was to develop an MPS to study cancer cell invasion in matrixes of different mechanical properties, mimicking, e.g., healthy or cancerous tissue. Here, the invasion of U3013 GBM cells was continuously monitored for 4 days of culture in a 2PP 3D printed hydrogel scaffold separating two microfluidic channels.

**FIG. 5.**
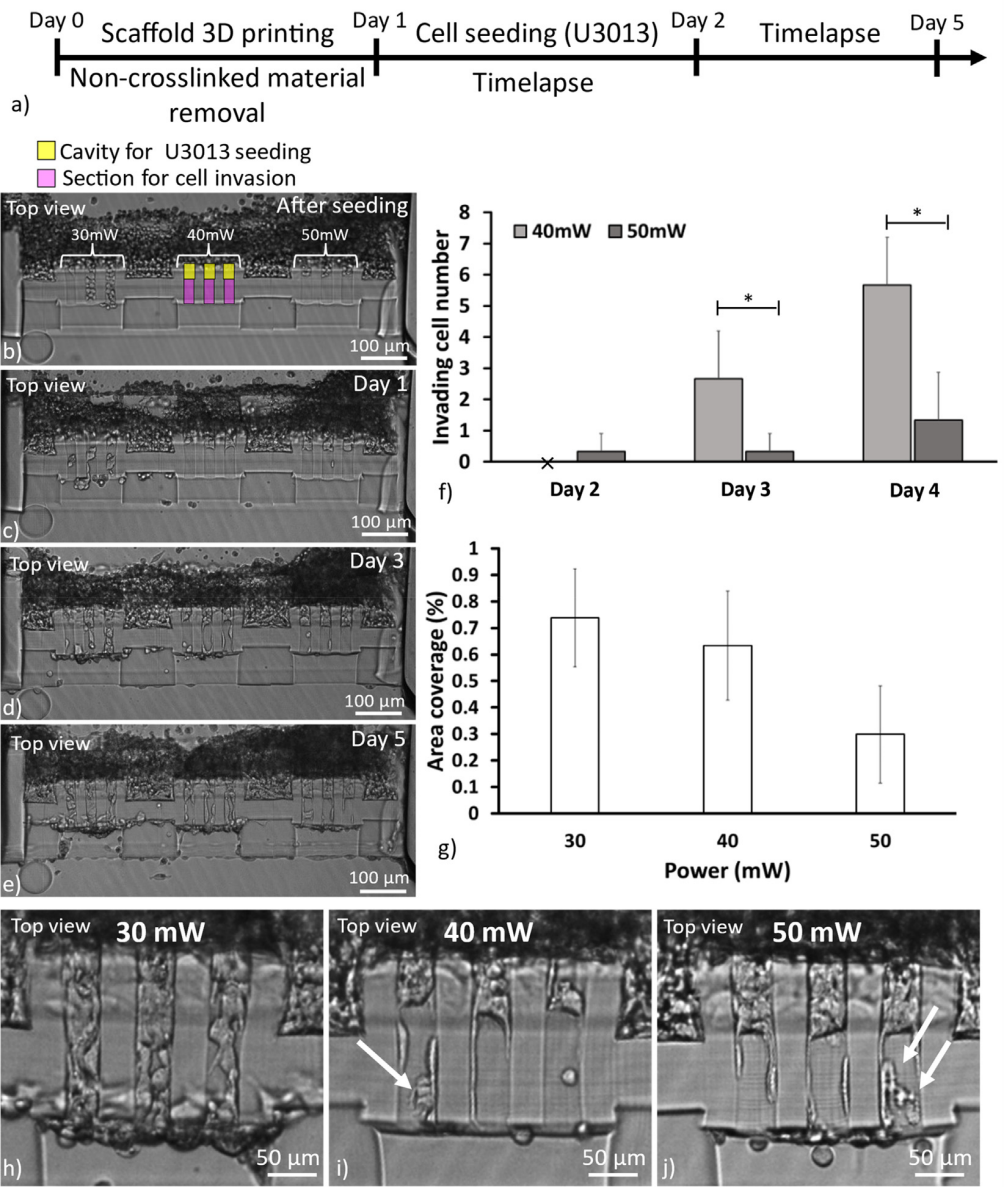
Scheme showing the scaffold 3D printing and the U3013 culture protocol (a). Brightfield images of U3013 cell invasion in the screened hydrogel over 5 days (b)–(e). The number of tumor cells invading the soft hydrogel section over 3 days of culture (f). * corresponds to significant differences between the samples at each time-point (p < 0.05, n = 3, error: standard deviation). The percentage of cell area coverage in the screened power sections (30, 40, and 50 mW) after 5 days of culture (n = 3, error: standard deviation) (g). Representative cell invasion patterns inside the sections obtained with 30 mW (h), 40 mW (i), and 50 mW (j), after 3 days of culture. The arrows indicate the influence of the hydrogel woodpile structure over the cell body during the cell invasion (Etaluma, 10× and 0.3 NA objective).

Tumor cells were seeded on one side of the scaffold and guided toward the migration sections by tilting the microfluidic device 90° for 2 h immediately after the cell solution injection, as in Figs. 5(a)–5(d). We opted for a high cell concentration (8 × 10^6^ cells/ml, volume injection:10 *μ*l) to ensure filling inside the scaffold cavities, as in Fig. 5(a).

After seeding, the sample was left in the incubator and cell migration was studied with time-lapse over the 5 days of culture (image interval: 30 min).

In the regions defined with 30 mW laser power, cells were immediately observed on both sides indicating that no hydrogel was present, as in Fig. 5(a), as previously observed in Sec. II A. We hypothesize that even if the sample appeared visible after printing, the poor adhesion between the fibers caused the structure to be unstable and be washed away during removal of the uncrosslinked hydrogel.

GBM cell invasion through the hydrogel matrix was observed for both regions crosslinked with 40 and 50 mW laser power, while no cells were observed to penetrate the support regions exposed at 90 mW, as shown in Fig. 5(d). During the first 48 h, no invasion was observed in the screening sections except for one cell in the 50 mW section. During day 2, the first cells started invading the hydrogel and reaching the other side of the scaffold, supplementary material, Video 2. The cell invasion of the hydrogel was then observed throughout the rest of the experiment. The 40 mW section displayed a higher invasiveness with a higher number of cells entering the screening section than for the 50 mW section, as in Figs. 5(d) and 5(e). The cell invasion was measured until the end of day 4 when the experiment was terminated as the presence of tumor cells on the other side of the scaffold interfered with the invasion of new cells, supplementary material, Video 2. Fabrication of longer sections (length above 75 *μ*m and diameter above 40 *μ*m) would have allowed longer monitoring times of the cell migration and simplified cell segmentation for analysis. However, the fabrication of bigger scaffolds would require longer 3D printing sessions representing a limitation due to the short printing window of the hydrogel precursor solution (3 h). In addition, bigger structures might affect the design capability to compensate for the swelling effect.

Previous studies have shown that cell motility is influenced by material porosity and stiffness^55^ and our results are in line with previous work that has reported on higher GBM cell invasiveness in scaffolds with low Young's modulus.^56,57^

We could also observe that the physical properties of the hydrogel affected the invasion pattern of the cells inside the hydrogel matrix where the morphology of the cells in the 50 mW exposed section was seen to be affected by the fiber arrangements of the hydrogel, as in Figs. 5(h) and 5(i). The tumor cells displayed a mesenchymal morphology, representative of GBM invasion,^58,59^ with protrusion extensions in the direction of invasion aligned to the fiber orientation. During the cell infiltration, the cell body shown confinement in the fiber grid architecture, indicating scaffold influence over the cell invasion, shown in Fig. 5(i). A similar effect, although less pronounced, was observed for the sections exposed to 40 mW, as in Fig. 5(h). Matrix architecture influence over the cell invasion was observed in previous *in vitro* studies for natural fiber-like hydrogels, i.e., collagen,^60^ *in vivo* and *ex vivo* with the consequence of a significant change in the tumor cell shape.^61,62^

Over time, a reduced constrain of the cells to the structure pattern was observed suggesting the tumor cell capability to remodel the ECM of the scaffold correlated with the metalloproteinase sites presence on the gelatin polymer chain,^46,63^ supplementary material, Video 2. After 5 days of culture, the 40 mW section displayed a higher area coverage than the 50 mW region suggesting a higher infiltration of tumor cells, as in Fig. 5(g).

The hydrogel scaffold design confined the majority of the swelling effect to the designated sections successfully separating the two microfluidic channels and enabling the study of cell invasiveness in hydrogels patterned using different laser powers. No cell invasion was observed outside the designated sections underlining the capability of the proposed fabrication strategy to spatially confine the cell invasion, as in Fig. 5(d). 40 mW laser power was chosen for the following experiments, focused on studying effects of cell–cell communication, as it allowed for the highest invasiveness.

### E. Interaction studies of endothelial and tumor cells on-chip

The behavior of tumor cells is not only influenced by the ECM matrix but also by their interaction with neighboring cells.^48^ An illustration of this is the vascular system that provides the tumor mass with nutrients and oxygen and creates a two-way cellular communication pathway. This interaction promotes cancer cells to invade the ECM and interact with blood vessels, facilitating their local and distant dissemination.^64–66^ GBM invasion, primarily observed in the brain, benefits from the high vascularization of the brain.^67^ In particular, chemoattractants secreted by the endothelial cells increase the invasiveness into the healthy brain regions.^68^

**FIG. 6.**
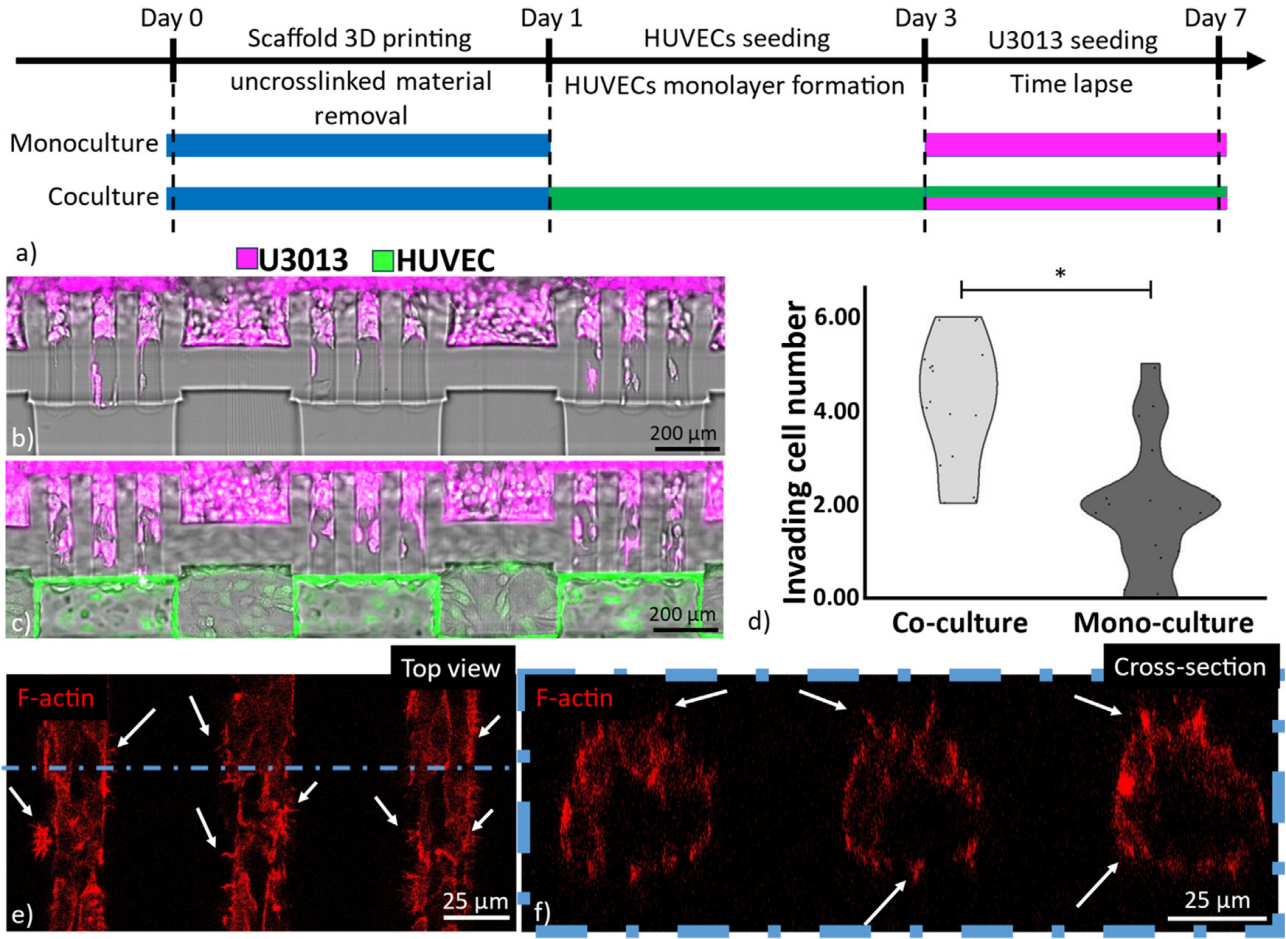
Tumor invasion comparison of U3013 monoculture and U3013-HUVEC co-culture. Scheme showing the scaffold 3D printing and the cell coculture protocol (a). U3013 cell invasion after 12 h of monoculture (b). U3013 invasion after 12 h of U3013-HUVEC co-culture (Leica SP8, 10× and 0.3 NA objective) (c). The number of cells invading the hydrogel channel after 12 h of cell culture. * corresponds to significant differences between the samples (p < 0.05, n = 18, error: standard deviation) (d). Top (e) and cross section (f); view of GBM cells invading the 40 mW sections in a coculture system after 4 days (Leica SP8, 25× and 0.95NA objective). The arrows indicate GBM filopodia; red: F-actin.

In this work, we explored the endothelium–tumor cell interaction by seeding GFP-HUVECs and fluorescently-labeled GBM cells on opposite sides of the hydrogel scaffold, shown in Fig. 6(b). HUVECs, previously used for glioblastoma studies, were chosen for this experiment.^69–71^ The ECM regions were obtained with a 40 mW laser power to ensure the highest invasiveness of the GBM cells. The cell loading into the MPS started with GFP-HUVEC seeding and culture for 2–3 days to obtain a confluent layer on one side of the hydrogel scaffold mimicking the vessel wall of the brain vasculature. Once this layer was formed, tumor cells were seeded, supplementary material, Video 3. We opted for sequential seeding to prevent the tumor cells from interfering with the GFP-HUVEC monolayer formation. The cell culture was performed for another 4 days in a stage top incubator (Okolab H301-K-FRAME) mounted on a confocal microscope (Leica SP8 10× 0.4 NA air objective) for optical monitoring of the two different cell types. The cell invasiveness of the co-culture was compared to the invasiveness of the tumor cells when cultured alone. For both conditions, HUVEC cell media was used, as shown by previous studies with GBM cells.^69,72,73^

Interestingly, a higher invasiveness during the first 12 h was observed for the tumor cells when they were co-cultured with GFP-HUVECs compared to the tumor cell monoculture, as in Fig. 6(c). Our results are in accordance with previous studies indicating an increase in tumor cell invasion in the presence of the endothelial cells.^74–76^

Despite the cell co-culture displaying a high number of U3013 cells invading the soft hydrogel section, a smaller amount of tumor cells reached the channel on the other side of the scaffold when compared to the monoculture. The reduced capability of the GBMs to access the opposite side of the hydrogel was associated with the presence of a barrier formed by the GFP-HUVECs, shown in supplementary material, Fig. 4.

Confocal microscopy imaging of cell co-culture displayed the low energy (40 mW) sections colonized by GBM cells after 4 days, shown in Fig. 6(d). The confinement of the cells inside the 40 mW cell invasion channel could be confirmed as no invasion was observed outside the designated invasion region. A non-uniform distribution of the tumor cells was observed with a higher presence of GBM at the interface between the 40 mW and 90 mW exposed sections, supplementary material, Video 4. No radial chemoattractants, oxygen, or nutrient gradients across the invasion section were identified as the cause of such GBM invasion pattern. It was hypothesized that the 90 mW-exposed hydrogel mechanically stabilized the peripheral area of the 40mW-exposed section providing a more stable fiber matrix for the GBM cells to invade the matrix.

Despite no invasion being observed in the parts of the scaffold printed with 90 mW laser power, the GBM presented F-actin-rich structures that radially spread inside the high energy crosslinked sections, as shown in Figs. 6(d) and 6(e). These structures were correlated with cell filopodia, involved in cell adhesion and motion. Interestingly, a lower density and size of filopodia per cell were observed for the HUVECs when compared to the U3013s indicating a lower penetration capability of the endothelial cells, as in supplementary material, Fig. 5.

The proposed MPS enabled time-lapse confocal imaging to monitor dynamics of U3013-HUVEC interactions in real time over 4 days of culture. The presence of the HUVEC monolayer initially hampered the invasion of the tumor cells in the vascular channel. However, GBM cells were observed migrating at the interface between the hydrogel and the HUVEC monolayer leading to the dissemination of the tumor cells along the hydrogel-HUVEC interface, supplementary material, Video 5. The tumor cell migration along the vasculature (vascular co-option) is a well reported strategy of tumor invasion in highly vascularized organs, i.e., brain.^68,77^ In particular, the perivascular spread of GBM represents a highly frequent invasion route^47,78^ considered as one of the causes of tumor resistance to treatments and tumor recurrence.^79^

**FIG. 7.**
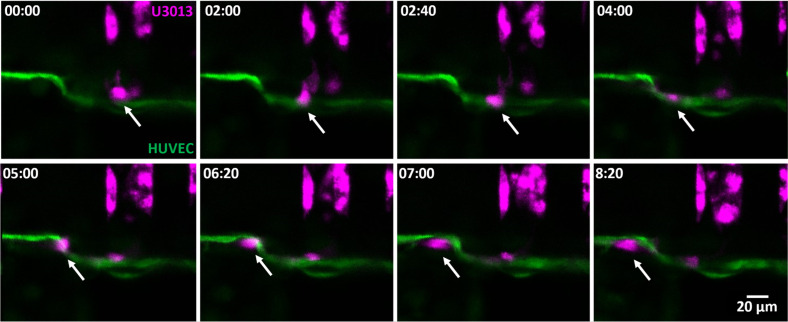
Time-lapse of a U3013 (magenta) reaching and migrating along the endothelial monolayer with penetration in the HUVEC barrier (green) between time point 05:00 and 06:20. Top view, time format (hh:mm).

As the amount of U3013 cells in contact with the HUVEC monolayer increased, GBM intravasation, after tumor invasion along the endothelial cells, was also observed, as in Fig. 7 and supplementary material, Video 6. The GBM filopodia interaction with the HUVEC monolayer continuity was also monitored, supplementary material, Videos 7(a) and 7(b). It has been reported that 39% of patients with diagnosed GBM have circulating tumor cells in the blood stream.^80^ However, this percentage poorly correlates with the extracranial metastasis frequency, between 0.4% and 2%.^80,81^ A possible answer to this paradox might lie in the high aggressiveness of the GBM with a median survival time below 15 months for a patient receiving treatments.^82^ A time interval considered insufficient for the development and detection of metastasis, as observed in patients only after 16–24 months following the initial tumor diagnosis.^81,83^ The limited life expectancy of the patients may account for the low incidence of metastasis due to the insufficient time for extracranial tumor development.

The previously reported capability of GBM to disrupt the blood-brain barrier places the intravasation as a possible cause of circulating tumor cells in the blood stream.^84^ Consequently, extracranial GBM metastasis may become an issue of clinical relevance as life expectancy of patients increases due to the development of more efficient GBM treatments.^46,83,85^

Four-day post co-culture, different distinguishing events of GBM-HUVEC interactions were observed, including GBM invasion, GBM migration along the endothelium layer, GBM intravasation, and endothelial monolayer recovery, supplementary material, Video 8.

The capability of capturing a high amount of cell–cell and cell–matrix interactions is fundamental to unravel the different mechanisms of tumor progression and the rare events due to rarity of incidence or difficulty to detect them. A small fraction of GBM cells was observed to fuse with endothelial cells (less than 1%).^86^ However, fusion between tumor cells and the surrounding cells has been reported as a cause to the tumor population diversity^87^ enhancing invasion capacity and the development of treatment resistance.^88^ During the co-culture, an event of tumor-HUVEC cell co-localization was visualized by observing the simultaneous motion of cells, suggesting cell fusion, supplementary material, Video 9.

The unique ability within our platform to tune the hydrogel properties combined with the possibility of imaging the dynamic interaction between endothelial and tumor cells in real time over extended time periods provides an important platform to gather valuable insights into biological events with significant potential for understanding, e.g., tumor invasion mechanisms.

## III. CONCLUSION

In the presented work, 2PP 3D printing technology was used to create a tumor-endothelium interaction model on-chip. We utilized the versatility of 2PP 3D printing to integrate this scaffold in a microfluidic chip with a simple geometry suitable for cleanroom-free fabrication. HUVEC and GBM cells were seeded on opposite sides of the hydrogel scaffold mimicking the ECM for investigating GBM invasion and interaction with the endothelial monolayer. The microfluidic device design allowed us to monitor tumor-endothelial cell interaction over 4 days demonstrating that the tumor cells more rapidly invaded the hydrogel matrix in the presence of an endothelial layer. Additionally, it was demonstrated that the physical properties of the hydrogel could be tuned during fabrication and, in turn, affect the tumor cell invasiveness. The robustness and versatility of the proposed system offer a valid platform for studying invasion of tumor cells and tumor–endothelial cell interactions in scaffolds with different mechanical properties mimicking, e.g., different tissues to increase our understanding of cancer progression.

## IV. METHODS

### A. 2PP setup

SolidWorks (Dassault systems) served to design and generate the standard triangle language (STL) file of all the 3D printed structures. The STL file was imported into the Think 3D software (UpNano GmbH) to set the printing parameters and allow the alignment of the structure to the microfluidic channel. Top-down mode and alternating adjacent layer scanning in *x* and *y* were chosen to improve the printing quality and reduce the asymmetric mechanical properties in the structures. For the hydrogel precursor solution, the refractive index of 1.36 was set as suggested by the ink supplier (BIO-INX). The structures were printed with the UpNano NanoOne_250_ 3D printer (UpNano GmbH) with a 10× objective (0.4 NA, Olympus) starting the print at the hydrogel–glass interface at the chip top.

### B. Microfluidic device fabrication

All the hydrogel manipulations were performed inside a polydimethylsiloxane (PDMS)-glass microfluidic chip. The glass and PDMS layers were air-plasma bonded (power: 200 W, time: 5 min, and model: Atto, Diener electronic GmbH) and then placed in contact on a hot plate at 100 °C for 2 h for bonding. Two different designs were used for (i) evaluating hydrogel swelling, performing FRAP experiments, and performing the cell experiment and (ii) investigating the hydrogel cytocompatibility for 2D culture of U3013 cells by using a device previously developed in our group.^31^ The layer-by-layer structure of device i) is displayed in supplementary material, Fig. 6(a).

### C. FRAP experiments

The printing parameter influence over the molecule diffusivity was investigated with 70 kDa FITC-Dextran (Sigma Aldrich) molecules at 50 *μ*M concentration. Five arrays of disks (100 *μ*m diameter and 120 *μ*m height) obtained with energy ranging from 30 to 100 mW were printed after manually injecting the hydrogel precursor solution into the microfluidic device with a pipette tip. After the printing, the microfluidic chip was immersed in cell media overnight to remove the non-crosslinked ink. Then, the hydrogel samples were incubated with a FITC-dextran solution for 24 h to reach equilibrium. The recovery of the bleached area was monitored for 30 s with 0.5 s frame interval with an inverted confocal microscope (Leica, SP8, 25× water objective, 0.95 NA). The recovery sequences were processed with the “FRAP analysis” library from MATLAB.^89^

### D. Hydrogel structure mean intensity, swelling, and fiber distance

Swelling of the 2PP ink at different energies was analyzed with a semiquantitative method previously used in studies for 2PP 3D printing.^49,60^ After 3D printing three arrays of disks (diameter 200 *μ*m and height 100 *μ*m) in the microfluidic chip, the samples were incubated in cell media for 24 h. The distance between neighboring disks was selected to avoid shadowing effects during 3D printing and stitching that causes artifacts due to the overlapping of adjacent printing tiles. The disk top structure surface area and mean intensity (*i.e.,* the surface furthest away from the hydrogel-glass interface) were measured with Cell Profiler after imaging via confocal microscopy.^90^ From the surface area, the disk diameter was calculated. The disk diameter was compared to the STL file dimension to determine the structure degree of swelling, according to the following formula:

$$\mathrm{Swelling}\;(\%)=\frac{D_{\mathrm{CAM}}-D_{\mathrm{CAD}}}{D_{\mathrm{CAD}}},$$

where *D_CAM_* is the diameter of the disk top and *D_CAD_* is the diameter of the CAD file.

Powers ranging from 30 to 100 mW were screened with constant hatching (0.5 *μ*m) and *z* intervals (3 *μ*m). A woodpile 3D printing method was set for all the samples. The distance between the fiber constituting the hydrogel structure was obtained by analyzing the intensity profile of the top surface of the disk via FIJI.^91^ The fluorescence (excitation around 405 nm and emission around 450 nm) of the printed hydrogel derives from the photoinitiator.^31^ The low energy section diameters were also measured by analyzing 10 cross sections of three sections obtained by the 30, 40, and 50 mW laser powers, respectively.

### E. Hydrogel preparation

A commercially available 2PP ink (U200, Bioinx) was used for all the 3D printed structures. The ink components were handled in a sterile environment and the hydrogel precursor solution was used within 3 h after mixing, as instructed by the supplier. To obtain the 2PP ink precursor solution, 7.5 *μ*l of the crosslinker solution (dithiothreitol, provided as ready-to-use solution by the supplier) was added to 50 *μ*l of the 2PP ink stock solution heated to 37 °C (provided as ready-to-use by the supplier) previously mixed with 42.5 *μ*l of PBS (Sigma Aldrich, concentration 1×). Potential air bubbles or debris were removed and collected at the bottom of the Eppendorf tube by centrifugation (100 g). Then, the solution was pipetted inside the microfluidic chip via one inlet of the fluidic device. To prevent contaminations and the dehydration of the solution, the inlets and outlets of the microfluidic chip were sealed with 3-mm punched PDMS disks (Superclear silicone sheet 0.5 *μ*m, Silex Silicones).

### F. Cell culture

Human umbilical vein endothelial cells (HUVEC) expressing green fluorescent protein (GFP) (Angio-Proteomie, PELOBiotech GmbH) were cultured in the suggested supplier cell culture medium (Cellovations, Endothelial Cell Growth Medium kit enhanced GFP, PELOBiotech). The culture flasks were coated for 1 h with a promoting cell adhesion solution (Speed Coating, PELOBiotech) prior to seeding cells. All HUVEC cells used in the experiments were between passages 4–10.

Patient-derived GBM cell line U3013 (www.hgcc.se) were cultured on laminin (10 *μ*g/ml) coated cell+ tissue culture flasks in serum-free neural stem cell (NSC) medium with FGF2 and EGF as described previously.^92^ Cells between passages 24 and 28 were used for the experiments.

All cell types were kept in culture in an incubator at 37 °C with 5% CO_2_ and saturated humidity. The cell culture medium was changed every 2 days.

### G. Mechanical investigation by nano indentation

Circular 2PP hydrogel disks (diameter: 1 mm and height: 250 *μ*m) were 3D printed on a glass slide. Nanoindentation characterization was performed using a Piuma Nanoindenter system (Optics 11) on day 2. The spherical indentation probe had a diameter of 24 *μ*m with a cantilever spring constant, k, of 0.025 N/m. Samples were immersed in PBS, and measurements were performed at an indentation depth of 1 *μ*m and displacement speed of 1 *μ*m/s with three samples and four repeats per sample for each condition. The Young's modulus was calculated using the built-in Piuma software by fitting force-indentation curves to the Hertzian contact mechanics model, assuming a Poisson ratio of 0.5 for incompressible materials.

### H. Cell culture on 2PP ink

The viability of U3013 cells was evaluated on a hydrogel disk (1 mm diameter and height 100 *μ*m) 3D printed at the bottom of the wells of device 2, following a previously developed protocol.^31^ The remaining non-crosslinked 2PP ink was removed by injecting fresh media in the microfluidic chip before the incubation for 24 h. For the cell viability test, the U3013 cells were seeded inside device 1 to obtain a cell density of 2 × 10^5^ cells/cm^2^. For the control, cells were cultured on a PO treated plate coated with laminin (10 *μ*g/ml). The cell viability was measured at 24 h after seeding (n = 3) by performing a live/dead assay with 0.75 *μ*l/ml propidium iodide (Thermo Fisher Scientific, P3566) and 2 *μ*l/ml Calcein AM (Thermo Fisher Scientific, C3009). After 15 min incubation of the injected assay solution, the microfluidic chip was washed three times with PBS. The stained cells were imaged by confocal microscope (Leica SP8 10× 0.4 NA air objective).

All the cultures of the cells on the hydrogel structure were performed in device 1, shown in supplementary material, Fig. 6. After printing the hydrogel scaffold, the remaining non-crosslinked hydrogel was washed out by injection of warm media and the microfluidic chip was incubated for 24 h. For evaluation of tumor cell invasion at different 3D printing energies (30–50 mW), a cell solution (8 × 10^6^ cells/ml) was injected in the channel inlet presenting the hydrogel scaffold cavities. Immediately after the solution injection, the microfluidic chip was tilted 90° in a 3D printed holder to ensure sedimentation of cells on the hydrogel scaffold. The construct was incubated for 1.5 h at the inclination. Following this, the microfluidic chip was placed in a 6 well plate, and the cells were monitored with an incubator microscope (Lumascope 560, Etaluma, 10X and 0.3 NA objective) set to acquire brightfield images with 10–30 min intervals. The U3013 invasion was determined by manually counting the cells invading the hydrogel area in the two sections printed at 40 and 50 mW laser power over the 3 days of culture. The final area coverage of the U3013 was manually measured after 4 days of culture.

For the cell coculture, U3013 cells were stained with Vybrant Dil (1/500) cell-labeling solution (Thermo Fisher Scientific) for 30 min in the cell culture flask at 37 °C inside an incubator. For the HUVECs and U3013 cells co-culture, the GFP-HUVEC solution (3 × 10^6^ cells/ml) was injected in the microfluidic channel opposite to the hydrogel cavities. After the formation of a monolayer (2–3 days), the stained U3013 cell solution (8 × 10^6^ cells/ml) was injected in the channel with the hydrogel cavities. For both seedings, the microfluidic chip was tiled 90° to ensure that the cells sedimented on the hydrogel scaffold. The cell culture was continuously monitored (20 min time lapse) inside a stage top incubator (Okolab H301-K-FRAME) mounted on a confocal microscope (Leica SP8 10× 0.4 NA air objective). The cell invasion in the channels was manually measured. At day 7, the samples with the cell cocultured were fixed with 2% paraformaldehyde for 15 min and stained for F-actin (Spirochrome Spy-actin 620 1 *μ*l/ml in PBS) for 1.5 h before three washes of PBS. The images were taken with a confocal microscope (Leica SP8, 25X water objective, 0.95 NA).

## SUPPLEMENTARY MATERIAL

See the supplementary material for additional information on the hydrogel scaffold design (Video S1 and Figs. S1 and S3), the microfluidic chip (Figs. S1 and S6), U3013-hydrogel interaction (Video S2), and U3013-HUVEC interaction (Video S3–S9, Figs. S3 and S5).

## Data Availability

The data that support the findings of this study are available from the corresponding author upon reasonable request.

## References

[1] F. Bray *et al.*, “ Global cancer statistics 2018: GLOBOCAN estimates of incidence and mortality worldwide for 36 cancers in 185 countries,” CA: Cancer J. Clin. 68, 394–424 (2018). 10.3322/caac.21492 30207593

[2] R. L. Siegel, K. D. Miller, H. E. Fuchs, and A. Jemal, “ Cancer statistics, 2022,” CA: Cancer J. Clin. 72, 7–33 (2022). 10.3322/caac.21708 35020204

[3] C. L. Chaffer and R. A. Weinberg, “ A perspective on cancer cell metastasis,” Science 331, 1559–1564 (2011). 10.1126/science.1203543 21436443

[4] C. Birzu *et al.*, “ Recurrent glioblastoma: From molecular landscape to new treatment perspectives,” Cancers 13(1), 47 (2020). 10.3390/cancers13010047 33375286 PMC7794906

[5] M. B. Chen *et al.*, “ On-chip human microvasculature assay for visualization and quantification of tumor cell extravasation dynamics,” Nat. Protoc. 12(5), 865–880 (2017). 10.1038/nprot.2017.018 28358393 PMC5509465

[6] A. F. Chambers, A. C. Groom, and I. C. MacDonald, “ Dissemination and growth of cancer cells in metastatic sites,” Nat. Rev. Cancer 2(8), 563–572 (2002). 10.1038/nrc865 12154349

[7] F. Fontana *et al.*, “ Requirements for animal experiments: Problems and challenges,” Small 17, 2004182 (2021). 10.1002/smll.202004182 33025748

[8] X. Liu *et al.*, “ Recent advances of organ-on-a-chip in cancer modeling research,” Biosensors 12, 1045 (2022). 10.3390/bios12111045 36421163 PMC9688857

[9] M. C. de Gooijer, M. Guillén Navarro, R. Bernards, T. Wurdinger, and O. van Tellingen, “ An experimenter's guide to glioblastoma invasion pathways,” Trends Mol. Med. 24, 763–780 (2018). 10.1016/j.molmed.2018.07.003 30072121

[10] H. H. Chung, M. Mireles, B. J. Kwarta, and T. R. Gaborski, “ Use of porous membranes in tissue barrier and co-culture models,” Lab Chip 18, 1671–1689 (2018). 10.1039/C7LC01248A 29845145 PMC5997570

[11] B. J. Kim and M. Wu, “ Microfluidics for mammalian cell chemotaxis,” Ann. Biomed. Eng. 40, 1316–1327 (2012). 10.1007/s10439-011-0489-9 22189490 PMC3424276

[12] S. R. Caliari and J. A. Burdick, “ A practical guide to hydrogels for cell culture,” Nat. Methods 13, 405–414 (2016). 10.1038/nmeth.3839 27123816 PMC5800304

[13] D. W. Grainger, “ Cell-based drug testing; this world is not flat,” Adv. Drug. Deliv. Rev. 69, vii–xi (2014). 10.1016/j.addr.2014.04.001 24709443

[14] K. Ronaldson-Bouchard and G. Vunjak-Novakovic, “ Organs-on-a-chip: A fast track for engineered human tissues in drug development,” Cell Stem Cell 22, 310–324 (2018). 10.1016/j.stem.2018.02.011 29499151 PMC5837068

[15] C. M. Leung *et al.*, “ A guide to the organ-on-a-chip,” Nat. Rev. Methods Primers 2, 33 (2022). 10.1038/s43586-022-00118-6

[16] C. C. Hsieh, S. B. Huang, P. C. Wu, D. B. Shieh, and G. B. Lee, “ A microfluidic cell culture platform for real-time cellular imaging,” Biomed. Microdevices 11, 903–913 (2009). 10.1007/s10544-009-9307-7 19370417

[17] A. P. Wong, R. Perez-Castillejos, J. Christopher Love, and G. M. Whitesides, “ Partitioning microfluidic channels with hydrogel to construct tunable 3-D cellular microenvironments,” Biomaterials 29, 1853–1861 (2008). 10.1016/j.biomaterials.2007.12.044 18243301 PMC2288785

[18] X. Mu, W. Zheng, J. Sun, W. Zhang, and X. Jiang, “ Microfluidics for manipulating cells,” Small 9, 9–21 (2013). 10.1002/smll.201200996 22933509

[19] W. Zhu, B. Holmes, R. I. Glazer, and L. G. Zhang, “ 3D printed nanocomposite matrix for the study of breast cancer bone metastasis,” Nanomedicine 12, 69–79 (2016). 10.1016/j.nano.2015.09.010 26472048

[20] N. Barin *et al.*, “ 3D-engineered scaffolds to study microtubes and localization of epidermal growth factor receptor in patient-derived glioma cells,” Small 18, 2204485 (2022). 10.1002/smll.202204485 36207287

[21] T. J. Kwak and E. Lee, “ In vitro modeling of solid tumor interactions with perfused blood vessels,” Sci. Rep. 10, 20142 (2020). 10.1038/s41598-020-77180-1 33214583 PMC7677310

[22] N. Walji, S. Kheiri, and E. W. K. Young, “ Angiogenic sprouting dynamics mediated by endothelial-fibroblast interactions in microfluidic systems,” Adv. Biol. 5, 2101080 (2021). 10.1002/adbi.202101080 34655165

[23] T. Miura and R. Yokokawa, “ Tissue culture on a chip: Developmental biology applications of self-organized capillary networks in microfluidic devices,” Dev. Growth Differ. 58, 505–515 (2016). 10.1111/dgd.12292 27272910

[24] D. T. T. Phan *et al.*, “ Blood–brain barrier-on-a-chip: Microphysiological systems that capture the complexity of the blood–central nervous system interface,” Exp. Biol. Med. 242, 1669 (2017). 10.1177/1535370217694100 PMC5786363 28195514

[25] D. S. Shin and K. S. Anseth, “ Recent advances in 3D models of tumor invasion,” Curr. Opin. Biomed. Eng. 19, 100310 (2021). 10.1016/j.cobme.2021.100310 34308009 PMC8294077

[26] M. P. Lutolf and J. A. Hubbell, “ Synthetic biomaterials as instructive extracellular microenvironments for morphogenesis in tissue engineering,” Nat. Biotechnol. 23, 47–55 (2005). 10.1038/nbt1055 15637621

[27] L. D. Harris, B. S. Kim, and D. J. Mooney, “ Open pore biodegradable matrices formed with gas foaming,” J. Biomed. Mater. Res. 42, 396–402 (1998). 10.1002/(SICI)1097-4636(19981205)42:3<396::AID-JBM7>3.0.CO;2-E 9788501

[28] Q. P. Pham, U. Sharma, and A. G. Mikos, “ Electrospun poly (ε-caprolactone) microfiber and multilayer nanofiber/microfiber scaffolds: Characterization of scaffolds and measurement of cellular infiltration,” Biomacromolecules 7, 2796–2805 (2006). 10.1021/bm060680j 17025355

[29] W. L. Murphy, R. G. Dennis, J. L. Kileny, and D. J. Mooney, “ Salt fusion: An approach to improve pore interconnectivity within tissue engineering scaffolds,” Tissue Eng. 8, 43 (2002). 10.1089/107632702753503045 11886653

[30] M. P. Cuchiara, A. C. B. Allen, T. M. Chen, J. S. Miller, and J. L. West, “ Multilayer microfluidic PEGDA hydrogels,” Biomaterials 31, 5491–5497 (2010). 10.1016/j.biomaterials.2010.03.031 20447685

[31] F. Cantoni, L. Barbe, H. Pohlit, and M. Tenje, “ A perfusable multi-hydrogel vasculature on-chip engineered by 2-photon 3D printing and scaffold molding to improve microfabrication fidelity in hydrogels,” Adv. Mater. Technol. 9, 2300718 (2024). 10.1002/admt.202300718

[32] A. Dobos *et al.*, “ On-chip high-definition bioprinting of microvascular structures,” Biofabrication 13, 015016 (2021). 10.1088/1758-5090/abb063 33586666

[33] S. Grebenyuk *et al.*, “ Large-scale perfused tissues via synthetic 3D soft microfluidics,” Nat. Commun. 14, 193 (2023). 10.1038/s41467-022-35619-1 36635264 PMC9837048

[34] A. Marino *et al.*, “ Magnetic self-assembly of 3D multicellular microscaffolds: A biomimetic brain tumor-on-a-chip for drug delivery and selectivity testing,” APL Bioeng. 7, 036103 (2023). 10.1063/5.0155037 37521177 PMC10375466

[35] A. Marino *et al.*, “ A 3D real-scale, biomimetic, and biohybrid model of the blood-brain barrier fabricated through two-photon lithography,” Small 14, 1702959 (2018). 10.1002/smll.201702959 PMC6715446 29239532

[36] A. Sharaf *et al.*, “ Two-photon polymerization of 2.5D and 3D microstructures fostering a ramified resting phenotype in primary microglia,” Front. Bioeng. Biotechnol. 10, 926642 (2022). 10.3389/fbioe.2022.926642 35979173 PMC9376863

[37] F. Cantoni *et al.*, “ Round-robin testing of commercial two-photon polymerization 3D printers,” Addit. Manuf. 76, 103761 (2023). 10.1016/j.addma.2023.103761

[38] S. G. Rayner *et al.*, “ Multiphoton-guided creation of complex organ-specific microvasculature,” Adv. Healthcare Mater. 10, e2100031 (2021). 10.1002/adhm.202100031 PMC8137585 33586357

[39] A. Ovsianikov, V. Mironov, J. Stampf, and R. Liska, “ Engineering 3D cell-culture matrices: Multiphoton processing technologies for biological and tissue engineering applications,” Expert Rev. Med. Devices 9, 613–633 (2012). 10.1586/erd.12.48 22943308

[40] J. Torgersen *et al.*, “ Hydrogels for two-photon polymerization: A toolbox for mimicking the extracellular matrix,” Adv. Funct. Mater. 23, 4542–4554 (2013). 10.1002/adfm.201203880

[41] N. Buch-Månson *et al.*, “ Rapid prototyping of polymeric nanopillars by 3D direct laser writing for controlling cell behavior,” Sci. Rep. 7(1), 1–9 (2017). 10.1038/s41598-017-09208-y 28835653 PMC5569057

[42] E. D. Lemma, B. Spagnolo, M. De Vittorio, and F. Pisanello, “ Studying cell mechanobiology in 3D: The two-photon lithography approach,” Trends Biotechnol. 37, 358–372 (2019). 10.1016/j.tibtech.2018.09.008 30343948

[43] B. Spagnolo *et al.*, “ Three-dimensional cage-like microscaffolds for cell invasion studies,” Sci. Rep. 5(1), 1–10 (2015). 10.1038/srep10531 PMC4650598 26013699

[44] J. Bauer, A. Guell Izard, Y. Zhang, T. Baldacchini, and L. Valdevit, “ Programmable mechanical properties of two-photon polymerized materials: From nanowires to bulk,” Adv. Mater. Technol. 4, 1900146 (2019). 10.1002/admt.201900146

[45] A. Sharaf, J. P. Frimat, G. J. Kremers, and A. Accardo, “ Suppression of auto-fluorescence from high-resolution 3D polymeric architectures fabricated via two-photon polymerization for cell biology applications,” Micro Nano Eng. 19, 100188 (2023). 10.1016/j.mne.2023.100188

[46] V. A. Cuddapah, S. Robel, S. Watkins, and H. Sontheimer, “ A neurocentric perspective on glioma invasion,” Nat. Rev. Neurosci. 15, 455–465 (2014). 10.1038/nrn3765 24946761 PMC5304245

[47] G. Seano and R. K. Jain, “ Vessel co-option in glioblastoma: Emerging insights and opportunities,” Angiogenesis 23, 9–16 (2020). 10.1007/s10456-019-09691-z 31679081 PMC7012982

[48] C. B. Crivii *et al.*, “ Glioblastoma microenvironment and cellular interactions,” Cancers 14, 1092 (2022). 10.3390/cancers14041092 35205842 PMC8870579

[49] A. Dobos *et al.*, “ Thiol–gelatin–norbornene bioink for laser-based high-definition bioprinting,” Adv. Healthcare Mater. 9, 1900752 (2020). 10.1002/adhm.201900752 31347290

[50] L. Brigo *et al.*, “ 3D high-resolution two-photon crosslinked hydrogel structures for biological studies,” Acta Biomater. 55, 373–384 (2017). 10.1016/j.actbio.2017.03.036 28351679

[51] D. Mandt *et al.*, “ Fabrication of biomimetic placental barrier structures within a microfluidic device utilizing two-photon polymerization,” Int. J. Bioprint. 4, 144 (2018). 10.18063/IJB.v4i2.144 33102920 PMC7581993

[52] J. A. W. van Dommelen, T. P. J. van der Sande, M. Hrapko, and G. W. M. Peters, “ Mechanical properties of brain tissue by indentation: Interregional variation,” J. Mech. Behav. Biomed. Mater. 3, 158–166 (2010). 10.1016/j.jmbbm.2009.09.001 20129415

[53] S. Budday *et al.*, “ Mechanical properties of gray and white matter brain tissue by indentation,” J. Mech. Behav. Biomed. Mater. 46, 318–330 (2015). 10.1016/j.jmbbm.2015.02.024 25819199 PMC4395547

[54] R. Stevens, L. Stevens, and N. C. Price, “ The stabilities of various thiol compounds used in protein purifications,” Biochem. Educ. 11(2), 70 (1983). 10.1016/0307-4412(83)90048-1

[55] M. Ehrbar *et al.*, “ Elucidating the role of matrix stiffness in 3D cell migration and remodeling,” Biophys. J. 100, 284–293 (2011). 10.1016/j.bpj.2010.11.082 21244824 PMC3021668

[56] C. Wang, X. Tong, and F. Yang, “ Bioengineered 3D brain tumor model to elucidate the effects of matrix stiffness on glioblastoma cell behavior using peg-based hydrogels,” Mol. Pharm. 11, 2115–2125 (2014). 10.1021/mp5000828 24712441

[57] T. J. Grundy *et al.*, “ Differential response of patient-derived primary glioblastoma cells to environmental stiffness,” Sci. Rep. 6, 23353 (2016). 10.1038/srep23353 26996336 PMC4800394

[58] J. Cha, S. G. Kang, and P. Kim, “ Strategies of mesenchymal invasion of patient-derived brain tumors: Microenvironmental adaptation,” Sci. Rep. 6, 24912 (2016). 10.1038/srep24912 27108713 PMC4842976

[59] G. M. O'Neill, J. Zhong, A. Paul, and S. J. Kellie, “ Mesenchymal migration as a therapeutic target in glioblastoma,” J. Oncol. 2010, 1. 10.1155/2010/430142 PMC2905941 20652056

[60] S. I. Fraley *et al.*, “ Three-dimensional matrix fiber alignment modulates cell migration and MT1-MMP utility by spatially and temporally directing protrusions,” Sci. Rep. 5(1), 1–13 (2015). 10.1038/srep14580 PMC4589685 26423227

[61] S. Watkins and H. Sontheimer, “ Hydrodynamic cellular volume changes enable glioma cell invasion,” J. Neurosci. 31, 17250–17259 (2011). 10.1523/JNEUROSCI.3938-11.2011 22114291 PMC3253353

[62] C. Beadle *et al.*, “ The role of myosin II in glioma invasion of the brain,” Mol. Biol. Cell 19, 3357–3368 (2008). 10.1091/mbc.e08-03-0319 18495866 PMC2488307

[63] K. Yue *et al.*, “ Synthesis, properties, and biomedical applications of gelatin methacryloyl (GelMA) hydrogels,” Biomaterials 73, 254–271 (2015). 10.1016/j.biomaterials.2015.08.045 26414409 PMC4610009

[64] L. Sevenich and J. A. Joyce, “ Pericellular proteolysis in cancer,” Genes Dev. 28, 2331 (2014). 10.1101/gad.250647.114 25367033 PMC4215179

[65] G. J. Baker *et al.*, “ Mechanisms of glioma formation: Iterative perivascular glioma growth and invasion leads to tumor progression, VEGF-independent vascularization, and resistance to antiangiogenic therapy,” Neoplasia 16, 543–561 (2014). 10.1016/j.neo.2014.06.003 25117977 PMC4198934

[66] S. Watkins *et al.*, “ Disruption of astrocyte-vascular coupling and the blood-brain barrier by invading glioma cells,” Nat. Commun. 5, 4196 (2014). 10.1038/ncomms5196 24943270 PMC4127490

[67] F. Seker-Polat, N. P. Degirmenci, I. Solaroglu, and T. Bagci-Onder, “ Tumor cell infiltration into the brain in glioblastoma: From mechanisms to clinical perspectives,” Cancers 14, 443 (2022). 10.3390/cancers14020443 35053605 PMC8773542

[68] A. Farin *et al.*, “ Transplanted glioma cells migrate and proliferate on host brain vasculature: A dynamic analysis,” Glia 53, 799–808 (2006). 10.1002/glia.20334 16541395

[69] M. T. Ngo and B. A. Harley, “ The influence of hyaluronic acid and glioblastoma cell coculture on the formation of endothelial cell networks in gelatin hydrogels,” Adv. Healthcare Mater. 6, 1700687 (2017). 10.1002/adhm.201700687 PMC5719875 28941173

[70] Q. Akolawala *et al.*, “ Micro-vessels-like 3D scaffolds for studying the proton radiobiology of glioblastoma-endothelial cells co-culture models,” Adv. Healthcare Mater. 13, e2302988 (2024). 10.1002/adhm.202302988 PMC11468971 37944591

[71] J. Bae, M. H. Kim, S. Han, and S. Park, “ Development of tumor-vasculature interaction on chip mimicking vessel co-option of glioblastoma,” BioChip J. 17, 77–84 (2023). 10.1007/s13206-022-00090-z

[72] Z. Chen *et al.*, “ In vitro angiogenesis by human umbilical vein endothelial cells (HUVEC) induced by three-dimensional co-culture with glioblastoma cells,” J. Neurooncol. 92, 121–128 (2009). 10.1007/s11060-008-9742-y 19039523

[73] D. T. Nguyen, Y. Fan, Y. M. Akay, and M. Akay, “ Investigating glioblastoma angiogenesis using a 3D in vitro GelMA microwell platform,” IEEE Trans. Nanobiosci. 15, 289–293 (2016). 10.1109/TNB.2016.2528170 27046878

[74] S. Nagaraju, D. Truong, G. Mouneimne, and M. Nikkhah, “ Microfluidic tumor–vascular model to study breast cancer cell invasion and intravasation,” Adv Healthcare Mater. 7, 1701257 (2018). 10.1002/adhm.201701257 29334196

[75] H. Cui *et al.*, “ Engineering a novel 3D printed vascularized tissue model for investigating breast cancer metastasis to bone,” Adv. Healthcare Mater. 9, e1900924 (2020). 10.1002/adhm.201900924 PMC7297662 31846231

[76] Y. Chonan, S. Taki, O. Sampetrean, H. Saya, and R. Sudo, “ Endothelium-induced three-dimensional invasion of heterogeneous glioma initiating cells in a microfluidic coculture platform,” Integr. Biol. 9, 762–773 (2017). 10.1039/C7IB00091J 28752870

[77] S. Wang and A. C. Dudley, “ Vascular Co-option in the brain tumor microenvironment,” in *Biomarkers of the Tumor Microenvironment*, 2nd ed. ( Springer International Publishing, 2022), pp. 537–547.

[78] V. Montana and H. Sontheimer, “ Bradykinin promotes the chemotactic invasion of primary brain tumors,” J. Neurosci. 31, 4858–4867 (2011). 10.1523/JNEUROSCI.3825-10.2011 21451024 PMC3096850

[79] N. Charles and E. C. Holland, “ The perivascular niche microenvironment in brain tumor progression,” Cell Cycle 9, 3084–3093 (2010). 10.4161/cc.9.15.12710 PMC3040926 20714216

[80] J. P. Sullivan *et al.*, “ Brain tumor cells in circulation are enriched for mesenchymal gene expression,” Cancer Discov. 4, 1299–1309 (2014). 10.1158/2159-8290.CD-14-0471 25139148 PMC4221467

[81] P. Beauchesne, “ Extra-neural metastases of malignant gliomas: Myth or reality?,” Cancers 3, 461–477 (2011). 10.3390/cancers3010461 24212625 PMC3756372

[82] N. F. Brown *et al.*, “ Survival outcomes and prognostic factors in glioblastoma,” Cancers 14, 3161 (2022). 10.3390/cancers14133161 35804940 PMC9265012

[83] A. L. Waack, A. D. Bhavsar, M. R. Ranabothu, A. T. Hoyt, and J. L. Schroeder, “ Letter to the editor regarding ‘unusual extraneural metastasis of glioblastoma,’ ” Surg. Neurol. Int. 14, 302 (2023). 10.25259/SNI_580_2023 37680923 PMC10481798

[84] S. W. Schneider *et al.*, “ Glioblastoma cells release factors that disrupt blood-brain barrier features,” Acta Neuropathol. 107, 272–276 (2004). 10.1007/s00401-003-0810-2 14730455

[85] G. S. Stoyanov *et al.*, “ Extracranial glioblastoma metastasis: A neuropathological case report,” Cureus 15(3), e35803 (2023). 10.7759/cureus.35803 37025749 PMC10073898

[86] S. El Hallani *et al.*, “ Tumor and endothelial cell hybrids participate in glioblastoma vasculature,” Biomed. Res. Int. 2014, 1. 10.1155/2014/827327 PMC4017715 24868550

[87] T. F. C. S. Warner, “ Cell hybridization: An explanation for the phenotypic diversity of certain tumours,” Med. Hypotheses 1(1), 51–57 (1975). 10.1016/0306-9877(75)90042-0 1105094

[88] H. Zhang *et al.*, “ Cell fusion-related proteins and signaling pathways, and their roles in the development and progression of cancer,” Front. Cell Dev. Biol. 9, 809668 (2022). 10.3389/fcell.2021.809668 35178400 PMC8846309

[89] P. Jönsson, M. P. Jonsson, J. O. Tegenfeldt, and F. Höök, “ A method improving the accuracy of fluorescence recovery after photobleaching analysis,” Biophys. J. 95, 5334 (2008). 10.1529/biophysj.108.134874 18567628 PMC2586554

[90] M. R. Lamprecht, D. M. Sabatini, and A. E. Carpenter, “ CellProfiler^TM^: Free, versatile software for automated biological image analysis,” Biotechniques 42, 71–75 (2007). 10.2144/000112257 17269487

[91] J. Schindelin *et al.*, “ Fiji: An open-source platform for biological-image analysis,” Nat. Methods 9, 676–682 (2012). 10.1038/nmeth.2019 22743772 PMC3855844

[92] Y. Xie *et al.*, “ The human glioblastoma cell culture resource: Validated cell models representing all molecular subtypes,” EBioMedicine 2, 1351–1363 (2015). 10.1016/j.ebiom.2015.08.026 26629530 PMC4634360

